# Bis(oxotremorine) fumarate bis­(fumaric acid)

**DOI:** 10.1107/S2414314622003649

**Published:** 2022-04-07

**Authors:** Marilyn Naeem, Andrew R. Chadeayne, James A. Golen, David R. Manke

**Affiliations:** a University of Massachusetts Dartmouth, 285 Old Westport Road, North Dartmouth, MA 02747, USA; b CaaMTech, Inc., 58 East Sunset Way, Suite 209, Issaquah, WA 98027, USA; Purdue University, USA

**Keywords:** crystal structure, hydrogen bonding, alkynes, pyrrolidines, fumarates

## Abstract

The title compound, bis­(oxotremorine) fumarate bis­(fumaric acid) {systematic name: 1-[4-(2-oxopyrrolidin-1-yl)but-2-yn­yl]pyrrolidinium (2*E*)-but-2-ene­di­o­ate bis­[(2*E*)-but-2-enedioic acid]}, 2C_12_H_19_N_2_O^+^·C_4_H_2_O_4_^2−^·2C_4_H_4_O_4_, has a single oxotremorine monocation protonated at the pyrrolidine nitro­gen, one fumaric acid mol­ecule and half of a fumarate dianion in the asymmetric unit. The ions and fumaric acid mol­ecules are held together by N—H⋯O and O–H-⋯O hydrogen bonds in 40-membered rings with graph-set notation *R* ^6^_6_(40). The fumarate ions join these rings into infinite chains along [001].

## Abstract 2

In the title mol­ecular salt, 2C_12_H_19_N_2_O^+^·C_4_H_2_O_4_^2−^·2C_4_H_4_O_4_, the components are held together by N—H⋯O and O—H⋯O hydrogen bonds, forming chains along [001].

## Graphical abstract

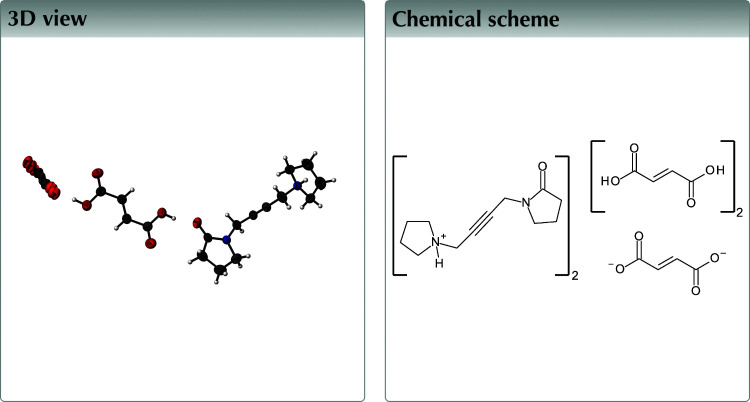

## Structure description

**Figure 1:**
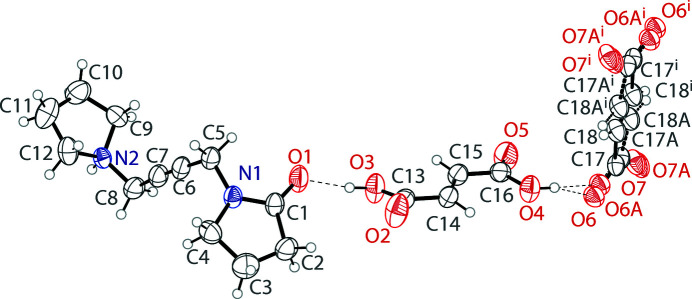
The mol­ecular structure of the title compound showing the atomic labeling. Displacement ellipsoids are drawn at the 50% probability level. Hydrogen bonds are shown as dashed lines. The asymmetric unit contains one half of a fumarate dianion, which is disordered over two positions. The other half of the inversion-generated fumarate dianion is shown. Symmetry code: (i) 2 − *x*, 1 − *y*, −*z*.

Oxotremorine is a selective agonist of the muscarinic acetyl­choline receptor, which reproduces many of the symptoms observed in Parkinson’s disease. This property has made it an invaluable tool in studying potential pharmaceuticals for Parkinson’s (Ringdahl & Jenden, 1983 ▸). A salt of oxotremorine that is commonly used in biological studies is produced by treating oxotremorine free base with fumaric acid. The resulting salt is reported as the sesquifumarate, indicating that the compound possesses an empirical formula with a 1:1.5 ratio of cation to fumarate dianion. However, the structure reported here shows that in the solid-state, the compound consists of two monocationic, protonated oxotremorines, one doubly deprotonated dianionic fumarate, and two fully protonated fumaric acid mol­ecules. One half of these ions and mol­ecules are present in the asymmetric unit (Fig. 1 ▸).

The only compound found by searching on ‘sesquifumarate’ in the Cambridge Structural Database (CSD, version 5.43, update of March 2022; Groom *et al.*, 2016 ▸) is that of the anti-arrhythmic agent tedisamil, which also exists as the bis­(cation) bis­(fumaric acid) fumarate and not the technical sesquifumarate (Jones *et al.*, 2004 ▸: CSD refcode EYOYUM). There are seven other bis­(cation) bis­(fumaric acid) fumarate salts (Haynes *et al.*, 2006 ▸: RESGEC, RESGUS; Provins *et al.*, 2006 ▸: SEGSAZ: Li & Zheng, 2005 ▸: QARKOK; Lin & Zheng, 2004 ▸: DAMYIA; Mohamed *et al.*, 2009 ▸: FUTNIS; Fang *et al.*, 2022 ▸: CCDC 2092690), and one bis­(cation) bis­(fumarate) fumaric acid salt (Collin *et al.*, 1987 ▸: FEMKIR) found in a search of the CSD. Although all of these structures incorporate three equivalents of fumaric acid into their structures relative to two cations, none is a formal sesquifumarate. The only such example in the CSD is that of Λ-cobalt(III) tris­(ethyl­enedi­amine), which shows all three fumaric acid mol­ecules to be fully deprotonated and in a 3:2 ratio with the tricationic cobalt complex ions (Liebig & Ruschewitz, 2012 ▸: PEJGAO). In general, there is a lack of precision when characterizing salts of fumaric acid, and diffraction studies are invaluable in distinguishing the different forms.

**Figure 2:**
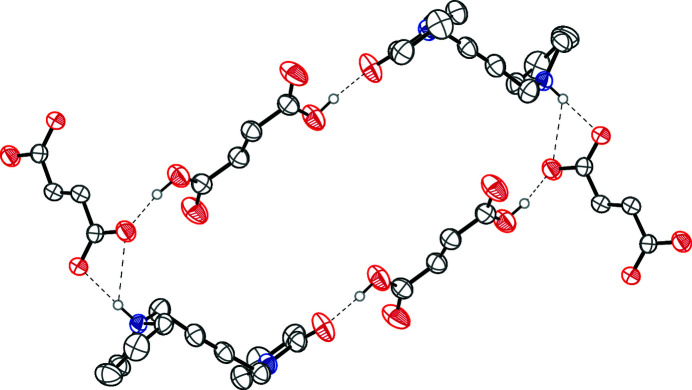
The hydrogen-bonding network forms chains along [001], which consist of 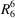 (40) rings that are joined together by the fumarate dianions. The ring structure is shown above. Hydrogen atoms not involved in hydrogen bonds, and the second component of the disordered fumarate dianion are omitted for clarity.

**Figure 3:**
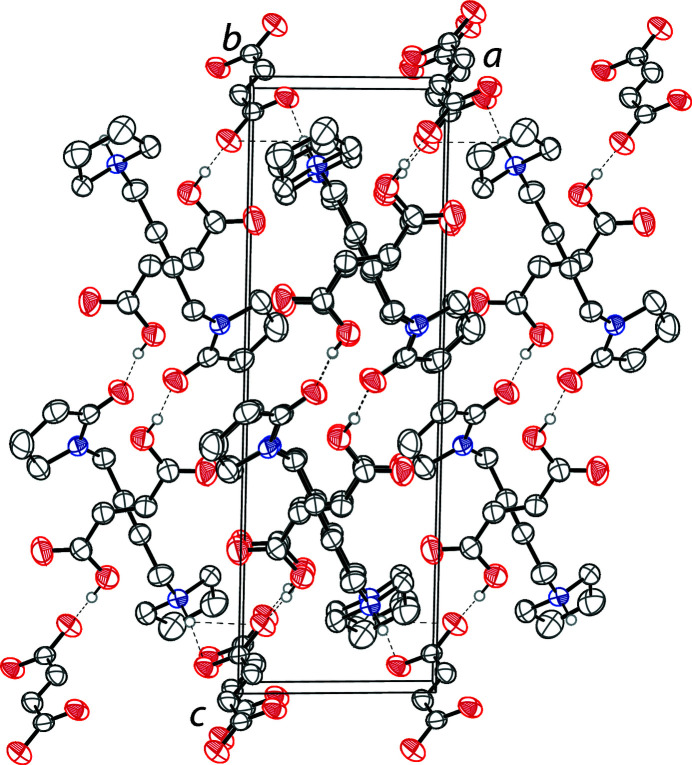
The crystal packing of the title compound viewed along the *b* axis. Hydrogen bonds are shown as dashed lines. Hydrogen atoms not involved in hydrogen bonds, and the second component of the disordered fumarate dianion are omitted for clarity.

In the structure of the title compound, the pyrrolidinium N—H of oxotremorine has bifurcated hydrogen bonds to two O atoms of a symmetry-generated fumarate dianion. One fumaric acid O—H hydrogen bonds to the carbonyl oxygen of the oxopyrrolidine of oxotremorine. The other fumaric acid O—H hydrogen bonds to one of the fumarate dianion oxygen atoms (Table 1 ▸). These hydrogen bonds connect two oxotremorine cations, two fumaric acid mol­ecules and two fumarate dianions into rings that have graph-set notation 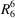 (40) (Etter *et al.*, 1990 ▸) (Fig. 2 ▸). The fumarate ions connect these rings together into infinite one-dimensional chains along [001]. The crystal packing of the title compound is shown in Fig. 3 ▸.

The fumaric acid and the fumarate dianion are near planar with r.m.s. deviations from planarity of 0.092 and 0.033 Å, respectively. The C—O distances of the fumarate mol­ecules are delocalized with values of 1.270 (3) and 1.243 (2) Å. The C—O distances in the fumaric acid mol­ecules are localized, with the carbonyl distances being 1.209 (2) and 1.203 (2) Å and the carbon–hydroxyl distances being 1.310 (2) and 1.316 (18) Å. The C—O distances and the location of the hydrogen atoms from the difference-Fourier map make the assignment of fumarate and fumaric acid clear.

In the reported structure, the amino­but-2-ynyl­ammonium unit has a near *anti* conformation, with a N2—C8—C5—N1 torsion angle of 163.17 (13)°. The other known structure of oxotremorine is reported as the sesquioxalate, but is similarly composed of the bis­(oxotremorine) bis­(oxalic acid) oxalate, and shows a torsion angle of 38.35 (3)° for the equivalent nitro­gen and carbon atoms (Clarke *et al.*, 1975 ▸: OXTREO). The other two similar structures reported, trimethyl-[4-(2-oxopyrrolidin-1-yl)but-2-yn­yl]-ammonium iodide (Baker & Pauling, 1973 ▸: MXPBYA), and a related acetyl­enic imidazole (Moon *et al.*, 1991 ▸: KOGCEO) show equivalent torsion angles of 143.50 (3) and 53.3 (4)°, respectively. The significant separation provided by the but-2-ynyl unit makes it so that there is no significant inter­action between the two units, giving no conformational preference.

## Synthesis and crystallization

Single crystals suitable for X-ray diffraction studies were grown by dissolving 15 mg of oxotremorine sesquifumarate purchased from Sigma–Aldrich in 5 ml of water. Solvent was allowed to evaporate at ambient temperature and pressure and crystals formed after 12 h.

## Refinement

Crystal data, data collection and structure refinement details are summarized in Table 2 ▸. The fumarate dianion is disordered over two positions (C17, C18, O6, O7 and C17*A*, C18*A*, O6*A*, O7*A*), which were modeled using a SAME restraint, as well as EADP instructions. The two components showed a 0.855 (4) to 0.145 (4) occupancy ratio.

## Supplementary Material

Crystal structure: contains datablock(s) I. DOI: 10.1107/S2414314622003649/zl4049sup1.cif

Structure factors: contains datablock(s) I. DOI: 10.1107/S2414314622003649/zl4049Isup2.hkl

Supporting information file. DOI: 10.1107/S2414314622003649/zl4049Isup3.cml

CCDC reference: 2163782

Additional supporting information:  crystallographic information; 3D view; checkCIF report

## Figures and Tables

**Table 1:** Hydrogen-bond geometry (Å, °)

*D*—H⋯*A*	*D*—H	H⋯*A*	*D*⋯*A*	*D*—H⋯*A*
C14—H14⋯O5^i^	0.93	2.65	3.498 (2)	152
C3—H3*B*⋯O1^i^	0.97	2.43	3.381 (3)	166
C5—H5*B*⋯O2^ii^	0.97	2.51	3.434 (2)	159
C8—H8*A*⋯O4^iii^	0.97	2.54	3.191 (2)	125
C8—H8*A*⋯O6^iii^	0.97	2.53	3.456 (3)	161
C8—H8*B*⋯O5^iv^	0.97	2.52	3.480 (2)	173
C9—H9*A*⋯O6^iv^	0.97	2.56	3.228 (3)	126
C10—H10*A*⋯O5^v^	0.97	2.66	3.624 (3)	175
C11—H11*A*⋯O4^vi^	0.97	2.64	3.553 (3)	157
C12—H12*B*⋯O7^vii^	0.97	2.68	3.399 (2)	132
C12—H12*B*⋯O7*A* ^vii^	0.97	2.37	3.139 (15)	135
O4—H4⋯O6	0.91 (1)	1.58 (1)	2.483 (2)	167 (2)
O3—H3⋯O1	0.90 (1)	1.69 (1)	2.5739 (16)	167 (2)
N2—H2⋯O6^iv^	0.908 (19)	2.554 (18)	3.172 (3)	125.8 (14)
N2—H2⋯O7^iv^	0.908 (19)	1.809 (19)	2.705 (2)	168.3 (17)
N2—H2⋯O6*A* ^iv^	0.908 (19)	2.43 (3)	3.131 (19)	133.6 (15)
N2—H2⋯O7*A* ^iv^	0.908 (19)	1.61 (2)	2.489 (15)	161.7 (17)

Symmetry codes: (i) 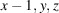 ; (ii) 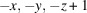 ; (iii) 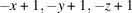 ; (iv) 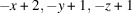 ; (v) 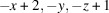 ; (vi) 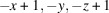 ; (vii) 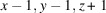 .

**Table 2:** Experimental details

Crystal data	
Chemical formula	2C_12_H_19_N_2_O^+^·C_4_H_2_O_4_^2−^·2C_4_H_4_O_4_
*M*_r_	760.78
Crystal system, space group	Triclinic, *P* 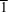
Temperature (K)	297
*a*, *b*, *c* (Å)	6.0921 (3), 8.5778 (5), 18.7260 (11)
α, β, γ (°)	94.922 (2), 90.428 (2), 98.945 (2)
*V* (Å^3^)	962.88 (9)
*Z*	1
Radiation type	Mo *K*α
μ (mm^−1^)	0.10
Crystal size (mm)	0.30 × 0.20 × 0.04

Data collection	
Diffractometer	Bruker D8 Venture CMOS
Absorption correction	Multi-scan (*SADABS*; Bruker, 2018 ▸)
*T*_min_, *T*_max_	0.717, 0.745
No. of measured, independent and observed [*I* > 2σ(*I*)] reflections	27417, 3651, 2975
*R*_int_	0.035
(sin θ/λ)_max_ (Å^−1^)	0.612

Refinement	
*R*[*F* ^2^ > 2σ(*F* ^2^)], *wR*(*F* ^2^), *S*	0.042, 0.109, 1.03
No. of reflections	3651
No. of parameters	269
No. of restraints	8
H-atom treatment	H atoms treated by a mixture of independent and constrained refinement
Δρ_max_, Δρ_min_ (e Å^−3^)	0.18, −0.15

Computer programs: *APEX3* (Bruker, 2018 ▸), *SAINT* (Bruker, 2018 ▸), *SHELXT2014* (Sheldrick, 2015*a* ▸), *SHELXL2018* (Sheldrick, 2015*b* ▸), *OLEX2* (Dolomanov *et al.*, 2009 ▸), and *publCIF* (Westrip, 2010 ▸).

## full crystallographic data

### Crystal data

**Table:**

2C_12_H_19_N_2_O^+^·C_4_H_2_O_4_^2^^−^·2C_4_H_4_O_4_	*Z* = 1
*M**_r_* = 760.78	*F*(000) = 404
Triclinic, *P*1	*D*_x_ = 1.312 Mg m^−^^3^
*a* = 6.0921 (3) Å	Mo *K*α radiation, λ = 0.71073 Å
*b* = 8.5778 (5) Å	Cell parameters from 9070 reflections
*c* = 18.7260 (11) Å	θ = 2.6–25.7°
α = 94.922 (2)°	µ = 0.10 mm^−^^1^
β = 90.428 (2)°	*T* = 297 K
γ = 98.945 (2)°	Block, colourless
*V* = 962.88 (9) Å^3^	0.30 × 0.20 × 0.04 mm

### Data collection

**Table:**

Bruker D8 Venture CMOS diffractometer	2975 reflections with *I* > 2σ(*I*)
φ and ω scans	*R*_int_ = 0.035
Absorption correction: multi-scan (SADABS; Bruker, 2018)	θ_max_ = 25.8°, θ_min_ = 2.6°
*T*_min_ = 0.717, *T*_max_ = 0.745	*h* = −7→7
27417 measured reflections	*k* = −10→10
3651 independent reflections	*l* = −22→22

### Refinement

**Table:**

Refinement on *F*^2^	Primary atom site location: structure-invariant direct methods
Least-squares matrix: full	Secondary atom site location: difference Fourier map
*R*[*F*^2^ > 2σ(*F*^2^)] = 0.042	Hydrogen site location: mixed
*wR*(*F*^2^) = 0.109	H atoms treated by a mixture of independent and constrained refinement
*S* = 1.03	*w* = 1/[σ^2^(*F*_o_^2^) + (0.0473*P*)^2^ + 0.2757*P*] where *P* = (*F*_o_^2^ + 2*F*_c_^2^)/3
3651 reflections	(Δ/σ)_max_ < 0.001
269 parameters	Δρ_max_ = 0.18 e Å^−^^3^
8 restraints	Δρ_min_ = −0.15 e Å^−^^3^

### Special details

**Table:**

Geometry. All esds (except the esd in the dihedral angle between two l.s. planes) are estimated using the full covariance matrix. The cell esds are taken into account individually in the estimation of esds in distances, angles and torsion angles; correlations between esds in cell parameters are only used when they are defined by crystal symmetry. An approximate (isotropic) treatment of cell esds is used for estimating esds involving l.s. planes.
Refinement. Hydrogen atoms H2, H3 and H4 were found from a difference-Fourier map and were refined isotropically, using *DFIX* restraints with O–H distances of 0.90 (1) Å. Isotropic displacement parameters were set to 1.2 *U*_eq_ of the parent nitrogen atom and 1.5 *U*_eq_ of the parent oxygen atom. All other hydrogen atoms were placed in calculated positions with C–H = 0.93 Å (*sp*^2^) or 0.97 Å (*sp*^3^). Isotropic displacement parameters were set to 1.2 *U*_eq_ of the parent carbon atom.

### Fractional atomic coordinates and isotropic or equivalent isotropic displacement parameters (Å^2^)

**Table:**

	*x*	*y*	*z*	*U*_iso_*/*U*_eq_	Occ. (<1)
O2	0.1880 (2)	0.32007 (19)	0.36008 (8)	0.0785 (5)	
O3	0.5181 (2)	0.34987 (18)	0.41423 (7)	0.0648 (4)	
O4	0.69301 (19)	0.57836 (15)	0.18323 (6)	0.0575 (3)	
O5	1.0269 (2)	0.56449 (19)	0.22771 (8)	0.0732 (4)	
C15	0.7126 (3)	0.45921 (19)	0.29059 (8)	0.0457 (4)	
H15	0.798508	0.415501	0.322444	0.055*	
C13	0.3847 (3)	0.36674 (19)	0.36123 (9)	0.0466 (4)	
C14	0.4977 (3)	0.44695 (18)	0.30158 (8)	0.0453 (4)	
H14	0.411762	0.491313	0.269994	0.054*	
C16	0.8265 (3)	0.53832 (18)	0.23060 (8)	0.0445 (4)	
O1	0.3504 (2)	0.16999 (17)	0.50962 (7)	0.0673 (4)	
N1	0.12900 (19)	0.08762 (16)	0.60010 (6)	0.0421 (3)	
N2	0.65707 (19)	0.00872 (15)	0.85784 (6)	0.0380 (3)	
C2	0.0229 (3)	0.2911 (2)	0.54231 (10)	0.0578 (5)	
H2A	−0.043039	0.281057	0.494497	0.069*	
H2B	0.094453	0.399740	0.553553	0.069*	
C1	0.1861 (2)	0.17907 (19)	0.54731 (8)	0.0445 (4)	
C3	−0.1513 (3)	0.2442 (3)	0.59673 (12)	0.0758 (6)	
H3A	−0.168967	0.334814	0.629641	0.091*	
H3B	−0.293254	0.203854	0.572933	0.091*	
C4	−0.0716 (3)	0.1172 (2)	0.63662 (10)	0.0576 (4)	
H4A	−0.039682	0.153881	0.686613	0.069*	
H4B	−0.181912	0.022144	0.633910	0.069*	
C5	0.2587 (3)	−0.02922 (19)	0.62190 (8)	0.0458 (4)	
H5A	0.370750	−0.043844	0.586468	0.055*	
H5B	0.162152	−0.129865	0.623917	0.055*	
C6	0.3680 (2)	0.01937 (19)	0.69249 (8)	0.0453 (4)	
C7	0.4569 (3)	0.0611 (2)	0.74894 (9)	0.0476 (4)	
C8	0.5696 (3)	0.1275 (2)	0.81681 (9)	0.0526 (4)	
H8A	0.466071	0.177765	0.846459	0.063*	
H8B	0.691969	0.208802	0.806785	0.063*	
C9	0.8224 (3)	−0.0784 (2)	0.82116 (8)	0.0463 (4)	
H9A	0.963732	−0.010026	0.817488	0.056*	
H9B	0.769788	−0.122425	0.773541	0.056*	
C10	0.8423 (3)	−0.2081 (3)	0.86974 (11)	0.0690 (5)	
H10A	0.867468	−0.304045	0.841843	0.083*	
H10B	0.964896	−0.175271	0.903809	0.083*	
C11	0.6245 (3)	−0.2359 (3)	0.90830 (12)	0.0729 (6)	
H11A	0.548597	−0.343053	0.896019	0.087*	
H11B	0.649915	−0.220532	0.959802	0.087*	
C12	0.4880 (3)	−0.1170 (2)	0.88405 (9)	0.0561 (4)	
H12A	0.383755	−0.164457	0.845940	0.067*	
H12B	0.406420	−0.074741	0.923545	0.067*	
H4	0.773 (3)	0.632 (3)	0.1495 (10)	0.089 (7)*	
H3	0.440 (4)	0.291 (3)	0.4458 (11)	0.098 (8)*	
H2	0.724 (3)	0.068 (2)	0.8967 (10)	0.053 (5)*	
O6	0.8934 (4)	0.7598 (3)	0.10050 (12)	0.0504 (5)	0.855 (4)
O7	1.1822 (4)	0.7858 (2)	0.02939 (11)	0.0519 (4)	0.855 (4)
C17	1.0122 (5)	0.7065 (2)	0.05155 (12)	0.0381 (5)	0.855 (4)
C18	0.9417 (3)	0.5389 (2)	0.02174 (10)	0.0442 (5)	0.855 (4)
H18	0.804798	0.487027	0.035288	0.053*	0.855 (4)
O6A	0.939 (3)	0.737 (2)	0.0861 (11)	0.0504 (5)	0.145 (4)
O7A	1.239 (2)	0.8253 (18)	0.0278 (9)	0.0519 (4)	0.145 (4)
C17A	1.096 (3)	0.7175 (18)	0.0433 (9)	0.0381 (5)	0.145 (4)
C18A	1.074 (2)	0.5595 (14)	0.0004 (6)	0.0442 (5)	0.145 (4)
H18A	1.189497	0.548224	−0.030918	0.053*	0.145 (4)

### Atomic displacement parameters (Å^2^)

**Table:**

	*U*^11^	*U*^22^	*U*^33^	*U*^12^	*U*^13^	*U*^23^
O2	0.0462 (7)	0.1052 (12)	0.0848 (10)	−0.0063 (7)	−0.0006 (6)	0.0461 (9)
O3	0.0503 (7)	0.0919 (10)	0.0556 (7)	0.0070 (6)	0.0046 (6)	0.0340 (7)
O4	0.0508 (7)	0.0692 (8)	0.0536 (7)	0.0034 (6)	0.0024 (5)	0.0224 (6)
O5	0.0460 (7)	0.0988 (11)	0.0808 (9)	0.0140 (7)	0.0119 (6)	0.0357 (8)
C15	0.0460 (8)	0.0463 (9)	0.0464 (9)	0.0088 (7)	0.0000 (7)	0.0097 (7)
C13	0.0450 (9)	0.0450 (9)	0.0518 (9)	0.0094 (7)	0.0042 (7)	0.0119 (7)
C14	0.0485 (9)	0.0434 (8)	0.0455 (8)	0.0073 (7)	0.0010 (7)	0.0118 (7)
C16	0.0447 (9)	0.0417 (8)	0.0481 (9)	0.0090 (7)	0.0054 (7)	0.0043 (7)
O1	0.0591 (7)	0.0944 (10)	0.0584 (8)	0.0248 (7)	0.0179 (6)	0.0389 (7)
N1	0.0367 (6)	0.0536 (8)	0.0374 (7)	0.0076 (5)	0.0000 (5)	0.0113 (6)
N2	0.0371 (6)	0.0446 (7)	0.0316 (6)	0.0043 (5)	−0.0009 (5)	0.0029 (5)
C2	0.0654 (11)	0.0583 (11)	0.0530 (10)	0.0192 (9)	−0.0103 (8)	0.0070 (8)
C1	0.0422 (8)	0.0542 (9)	0.0372 (8)	0.0050 (7)	−0.0054 (6)	0.0096 (7)
C3	0.0560 (11)	0.0972 (16)	0.0831 (14)	0.0325 (11)	0.0053 (10)	0.0203 (12)
C4	0.0504 (9)	0.0678 (11)	0.0563 (10)	0.0127 (8)	0.0130 (8)	0.0077 (8)
C5	0.0472 (8)	0.0510 (9)	0.0409 (8)	0.0088 (7)	−0.0014 (7)	0.0115 (7)
C6	0.0423 (8)	0.0526 (9)	0.0438 (9)	0.0090 (7)	0.0019 (7)	0.0178 (7)
C7	0.0446 (8)	0.0573 (10)	0.0439 (9)	0.0126 (7)	−0.0001 (7)	0.0138 (7)
C8	0.0626 (10)	0.0514 (10)	0.0463 (9)	0.0166 (8)	−0.0070 (8)	0.0060 (7)
C9	0.0411 (8)	0.0559 (10)	0.0412 (8)	0.0090 (7)	0.0030 (6)	−0.0020 (7)
C10	0.0741 (13)	0.0688 (13)	0.0702 (12)	0.0277 (10)	−0.0078 (10)	0.0112 (10)
C11	0.0741 (13)	0.0640 (12)	0.0794 (14)	−0.0056 (10)	−0.0103 (11)	0.0305 (11)
C12	0.0410 (8)	0.0748 (12)	0.0499 (9)	−0.0049 (8)	0.0030 (7)	0.0164 (8)
O6	0.0583 (12)	0.0436 (10)	0.0486 (13)	0.0053 (7)	0.0167 (7)	0.0044 (8)
O7	0.0575 (14)	0.0492 (13)	0.0408 (7)	−0.0149 (9)	0.0115 (9)	−0.0009 (9)
C17	0.0421 (14)	0.0411 (9)	0.0297 (10)	0.0013 (10)	−0.0020 (11)	0.0048 (7)
C18	0.0432 (10)	0.0447 (11)	0.0402 (10)	−0.0065 (8)	0.0062 (8)	0.0030 (8)
O6A	0.0583 (12)	0.0436 (10)	0.0486 (13)	0.0053 (7)	0.0167 (7)	0.0044 (8)
O7A	0.0575 (14)	0.0492 (13)	0.0408 (7)	−0.0149 (9)	0.0115 (9)	−0.0009 (9)
C17A	0.0421 (14)	0.0411 (9)	0.0297 (10)	0.0013 (10)	−0.0020 (11)	0.0048 (7)
C18A	0.0432 (10)	0.0447 (11)	0.0402 (10)	−0.0065 (8)	0.0062 (8)	0.0030 (8)

### Geometric parameters (Å, º)

**Table:**

O2—C13	1.2026 (19)	C5—H5A	0.9700
O3—C13	1.310 (2)	C5—H5B	0.9700
O3—H3	0.902 (10)	C5—C6	1.473 (2)
O4—C16	1.3016 (19)	C6—C7	1.185 (2)
O4—H4	0.914 (10)	C7—C8	1.467 (2)
O5—C16	1.2092 (19)	C8—H8A	0.9700
C15—H15	0.9300	C8—H8B	0.9700
C15—C14	1.316 (2)	C9—H9A	0.9700
C15—C16	1.483 (2)	C9—H9B	0.9700
C13—C14	1.480 (2)	C9—C10	1.515 (3)
C14—H14	0.9300	C10—H10A	0.9700
O1—C1	1.2372 (19)	C10—H10B	0.9700
N1—C1	1.3293 (19)	C10—C11	1.511 (3)
N1—C4	1.451 (2)	C11—H11A	0.9700
N1—C5	1.452 (2)	C11—H11B	0.9700
N2—C8	1.486 (2)	C11—C12	1.509 (3)
N2—C9	1.4833 (19)	C12—H12A	0.9700
N2—C12	1.490 (2)	C12—H12B	0.9700
N2—H2	0.908 (19)	O6—C17	1.270 (3)
C2—H2A	0.9700	O7—C17	1.243 (2)
C2—H2B	0.9700	C17—C18	1.492 (3)
C2—C1	1.495 (2)	C18—C18^i^	1.295 (4)
C2—C3	1.510 (3)	C18—H18	0.9300
C3—H3A	0.9700	O6A—C17A	1.273 (14)
C3—H3B	0.9700	O7A—C17A	1.222 (14)
C3—C4	1.509 (3)	C17A—C18A	1.502 (14)
C4—H4A	0.9700	C18A—C18A^i^	1.25 (2)
C4—H4B	0.9700	C18A—H18A	0.9300
			
C13—O3—H3	108.7 (16)	H5A—C5—H5B	107.9
C16—O4—H4	110.0 (15)	C6—C5—H5A	109.3
C14—C15—H15	117.9	C6—C5—H5B	109.3
C14—C15—C16	124.13 (15)	C7—C6—C5	178.83 (17)
C16—C15—H15	117.9	C6—C7—C8	174.81 (17)
O2—C13—O3	123.57 (15)	N2—C8—H8A	108.7
O2—C13—C14	122.35 (15)	N2—C8—H8B	108.7
O3—C13—C14	114.08 (14)	C7—C8—N2	114.07 (14)
C15—C14—C13	123.90 (15)	C7—C8—H8A	108.7
C15—C14—H14	118.1	C7—C8—H8B	108.7
C13—C14—H14	118.1	H8A—C8—H8B	107.6
O4—C16—C15	114.39 (13)	N2—C9—H9A	111.2
O5—C16—O4	123.90 (15)	N2—C9—H9B	111.2
O5—C16—C15	121.70 (15)	N2—C9—C10	102.94 (13)
C1—N1—C4	114.58 (13)	H9A—C9—H9B	109.1
C1—N1—C5	123.48 (13)	C10—C9—H9A	111.2
C4—N1—C5	121.90 (13)	C10—C9—H9B	111.2
C8—N2—C12	116.01 (13)	C9—C10—H10A	110.5
C8—N2—H2	103.0 (11)	C9—C10—H10B	110.5
C9—N2—C8	116.32 (12)	H10A—C10—H10B	108.7
C9—N2—C12	104.70 (13)	C11—C10—C9	106.06 (15)
C9—N2—H2	108.6 (11)	C11—C10—H10A	110.5
C12—N2—H2	107.8 (11)	C11—C10—H10B	110.5
H2A—C2—H2B	108.8	C10—C11—H11A	110.5
C1—C2—H2A	110.7	C10—C11—H11B	110.5
C1—C2—H2B	110.7	H11A—C11—H11B	108.7
C1—C2—C3	105.11 (15)	C12—C11—C10	106.27 (15)
C3—C2—H2A	110.7	C12—C11—H11A	110.5
C3—C2—H2B	110.7	C12—C11—H11B	110.5
O1—C1—N1	123.90 (15)	N2—C12—C11	103.58 (13)
O1—C1—C2	126.98 (15)	N2—C12—H12A	111.0
N1—C1—C2	109.11 (14)	N2—C12—H12B	111.0
C2—C3—H3A	110.4	C11—C12—H12A	111.0
C2—C3—H3B	110.4	C11—C12—H12B	111.0
H3A—C3—H3B	108.6	H12A—C12—H12B	109.0
C4—C3—C2	106.81 (15)	O6—C17—C18	116.8 (2)
C4—C3—H3A	110.4	O7—C17—O6	123.1 (2)
C4—C3—H3B	110.4	O7—C17—C18	120.1 (2)
N1—C4—C3	104.08 (14)	C17—C18—H18	117.8
N1—C4—H4A	110.9	C18^i^—C18—C17	124.4 (2)
N1—C4—H4B	110.9	C18^i^—C18—H18	117.8
C3—C4—H4A	110.9	O6A—C17A—C18A	116.1 (16)
C3—C4—H4B	110.9	O7A—C17A—O6A	123.5 (16)
H4A—C4—H4B	109.0	O7A—C17A—C18A	119.4 (16)
N1—C5—H5A	109.3	C17A—C18A—H18A	114.6
N1—C5—H5B	109.3	C18A^i^—C18A—C17A	130.7 (16)
N1—C5—C6	111.67 (13)	C18A^i^—C18A—H18A	114.6
			
O2—C13—C14—C15	−159.84 (18)	C5—N1—C1—O1	2.9 (2)
O3—C13—C14—C15	19.3 (2)	C5—N1—C1—C2	−176.77 (14)
C14—C15—C16—O4	−8.4 (2)	C5—N1—C4—C3	−179.65 (16)
C14—C15—C16—O5	170.45 (18)	C8—N2—C9—C10	−169.44 (14)
C16—C15—C14—C13	179.50 (15)	C8—N2—C12—C11	168.12 (15)
N2—C9—C10—C11	25.88 (19)	C9—N2—C8—C7	59.62 (19)
C2—C3—C4—N1	−4.8 (2)	C9—N2—C12—C11	38.48 (17)
C1—N1—C4—C3	2.4 (2)	C9—C10—C11—C12	−2.6 (2)
C1—N1—C5—C6	109.44 (16)	C10—C11—C12—N2	−21.6 (2)
C1—C2—C3—C4	5.5 (2)	C12—N2—C8—C7	−64.18 (19)
C3—C2—C1—O1	176.15 (18)	C12—N2—C9—C10	−39.98 (16)
C3—C2—C1—N1	−4.2 (2)	O6—C17—C18—C18^i^	−171.2 (3)
C4—N1—C1—O1	−179.16 (16)	O7—C17—C18—C18^i^	7.4 (4)
C4—N1—C1—C2	1.17 (19)	O6A—C17A—C18A—C18A^i^	−1 (3)
C4—N1—C5—C6	−68.36 (19)	O7A—C17A—C18A—C18A^i^	−169 (2)

Symmetry code: (i) −*x*+2, −*y*+1, −*z*.

### Hydrogen-bond geometry (Å, º)

**Table:**

*D*—H···*A*	*D*—H	H···*A*	*D*···*A*	*D*—H···*A*
C14—H14···O5^ii^	0.93	2.65	3.498 (2)	152
C3—H3*B*···O1^ii^	0.97	2.43	3.381 (3)	166
C5—H5*B*···O2^iii^	0.97	2.51	3.434 (2)	159
C8—H8*A*···O4^iv^	0.97	2.54	3.191 (2)	125
C8—H8*A*···O6^iv^	0.97	2.53	3.456 (3)	161
C8—H8*B*···O5^v^	0.97	2.52	3.480 (2)	173
C9—H9*A*···O6^v^	0.97	2.56	3.228 (3)	126
C10—H10*A*···O5^vi^	0.97	2.66	3.624 (3)	175
C11—H11*A*···O4^vii^	0.97	2.64	3.553 (3)	157
C12—H12*B*···O7^viii^	0.97	2.68	3.399 (2)	132
C12—H12*B*···O7*A*^viii^	0.97	2.37	3.139 (15)	135
O4—H4···O6	0.91 (1)	1.58 (1)	2.483 (2)	167 (2)
O3—H3···O1	0.90 (1)	1.69 (1)	2.5739 (16)	167 (2)
N2—H2···O6^v^	0.908 (19)	2.554 (18)	3.172 (3)	125.8 (14)
N2—H2···O7^v^	0.908 (19)	1.809 (19)	2.705 (2)	168.3 (17)
N2—H2···O6*A*^v^	0.908 (19)	2.43 (3)	3.131 (19)	133.6 (15)
N2—H2···O7*A*^v^	0.908 (19)	1.61 (2)	2.489 (15)	161.7 (17)

Symmetry codes: (ii) *x*−1, *y*, *z*; (iii) −*x*, −*y*, −*z*+1; (iv) −*x*+1, −*y*+1, −*z*+1; (v) −*x*+2, −*y*+1, −*z*+1; (vi) −*x*+2, −*y*, −*z*+1; (vii) −*x*+1, −*y*, −*z*+1; (viii) *x*−1, *y*−1, *z*+1.
